# Isolated duodenal perforation at D4 following blunt abdominal trauma

**DOI:** 10.1016/j.ijscr.2020.06.076

**Published:** 2020-06-20

**Authors:** Marilynn Omondi, Irene Mutua, Dan Kiptoon

**Affiliations:** University of Nairobi, Kenya

**Keywords:** Duodenal injury, Blunt abdominal trauma, Peritonitis, High index of suspicion, Case report

## Abstract

**Introduction:**

Isolated duodenal injuries are particularly rare in blunt abdominal trauma as the duodenum is at a deep and relatively well-protected anatomical site.

**Case presentation:**

We present a case report of a 22-year-old male patient who presented to an accident and emergency department at a tertiary training hospital within four hours of sustaining blunt abdominal trauma. His vital signs were stable at this point and was found to have slight abdominal tenderness in the epigastric area with no abdominal rigidity and normal bowel sounds on auscultation.

A CT Scan of the abdomen done was normal. He was admitted to the surgical ward for serial abdominal monitoring.

Eight hours into his admission, his physical condition deteriorated necessitating an emergency laparotomy where a perforation of D4 on the anterior wall was found. This was repaired primarily and he had a relatively calm post-operative stay in the surgical ward and was discharged home.

**Discussion:**

Diagnosis of blunt duodenal injury is often delayed because of its retroperitoneal nature. Initial clinical changes in isolated duodenal injury may be extremely subtle before peritonitis sets in. It is therefore important to consider both mechanism of injury and other clinical signs such as tachycardia and raised white cell count as delays in diagnosis and subsequent management adversely affect morbidity and mortality.

**Conclusion:**

Timely management of this rare and life threatening injury is hinged on a high index of suspicion in spite of what previous imaging may show to the surgeon.

## Highlights

• Isolated duodenal injuries are rare in blunt abdominal trauma.
• Proper diagnosis mandates a high clinical index of suspicion.
• Initial clinical changes may be extremely subtle before peritonitis sets in.
• CT scan of the abdomen is the diagnostic test of choice in stable patients.
• CT scan abdomen may be negative if done too early after the injury.

## 1 Introduction

Isolated duodenal rupture following blunt abdominal trauma is rare and represents approximately 2%–20% of patients with blunt abdominal trauma that occur after blows to the upper abdomen, or abdominal compression from high-riding seat belts [1]. Forty percent of patients with duodenal injuries have other concomitant intra-abdominal injuries, such as hepatic (38%), or pancreatic (28%) injuries [2]. It is likely that patients’ with concomitant injuries have lower morbidity and mortality as the associated injuries prompt the surgeon to intervene faster than in isolated injuries that initially have subtle signs that may be missed unless the surgeon has a high index of suspicion and is cognisant of the mechanism of trauma that can cause duodenal injuries. Ultrasound and CT scan form the mainstay of initial investigation in blunt abdominal trauma with the CT being much more detailed in anatomical demonstration. However if the CT scan is done too early after the injury it may be negative for isolated duodenal injury [3].

This case has been reported in line with the SCARE Criteria [4].

## 2 Case presentation

A 22-year-old male presented to the accident and emergency department at a tertiary training hospital with a history of having been involved in a road traffic accident. He was reported to have been an unrestrained driver of a vehicle that was involved in a head on collision approximately four hours prior to time of presentation.

The patient came in complaining of mild epigastric pain for 4 h. He was examined using the ATLS protocol. He was talking on presentation and he had no neck pain but still had a cervical collar applied. His breathing was not laboured and examination of the chest was normal. His vital signs were stable at this point in time with a Blood pressure of 110/80 mmHg, a pulse rate of 80 beats per minute and a respiratory rate of 16 breaths per minute. Positive findings were noted in the abdominal examination. He was found to have slight abdominal tenderness in the epigastric area with no abdominal rigidity and normal bowel sounds on auscultation. He had no flank ecchymosis or seat belt sign noted. Digital rectal examination was normal. His secondary survey revealed no further abdominal findings. His initial laboratory results were also normal. A chest radiograph did not show any evidence of free air under the diaphragm. A focused abdominal ultrasound for trauma (FAST) scan done showed minimal peri-hepatic fluid with no obvious solid organ injury. An abdominal Computed Tomography (CT) scan with intravenous contrast done was normal. The patient was admitted to the surgical ward and was started on analgesia, kept Nil Per Os (NPO) and was to have vital sign monitoring with serial four-hourly abdominal examinations.

Eight hours into his admission, he was noted to have a temperature of 39 °C, a blood pressure of 90/50 mmHg, and a pulse rate of 120 beats per minute. Physical examination revealed a diffusely tender abdomen with guarding and reduced bowel sounds. He also reported worsening epigastric pain that was not responding to analgesia with vomiting. Repeat blood works showed he had an elevated white cell count of 21.3 × 10^9^/l and normal serum amylase and lipase. In addition to the treatment he was getting he was also started on antibiotics and antipyretics. The patient was prepped for an emergency laparotomy and consent obtained for the same as he now had peritonitis.

At laparotomy, a perforation of D4 on the anterior wall was found involving less than 50% of the circumference with minimal abdominal contamination. No other injuries were noted and all solid organs were normal. The lacerated edges were freshened and repaired in a single layer using interrupted 3-0 polyglactin suture. An intra-abdominal drain was left in-situ.

Post operatively the patient was admitted to the High dependency unit. On post-operative day one, his blood pressure was 110/70 mmHg, a pulse rate of 90 beats per minute, respiratory rate of 18 breaths per minute and an SPO2 of 96% on room air with a urine output of 2mls/kg/hr. The drain on day one was minimally active with less than 50 ml noted. He was started on incentive chest spirometry and encouraged to start ambulation. Post-operative day two, his vital signs remained stable, He had no fever and was in less pain and was allowed to start feeding as he tolerated. He was already ambulating and the drain remained minimally active. His wound on examination remained clean and showed no features of infection. Blood works repeated 48 h post laparotomy, showed a drop in his white cell count to about 14.6 × 10^9^/l. His amylase and lipase remained normal. He was stepped down to the General surgical floor and was allowed to go home on the fifth post-operative day.

## 3 Discussion

The duodenum is a ‘C’-shaped organ situated in the retroperitoneal space and is anatomically divided into four sections (D1–D4). It is vulnerable to damage by shearing or compression forces with D1 and D4 being relatively mobile in comparison with D2 and D3 which are fixed. Commonly, injuries occur at the junction between D1 and D2 or at the junction between D3 and D4 [5,6]. Duodenal rupture following blunt abdominal trauma usually occurs after a road traffic accident and falls from a height [7,8]. These are commonly associated with injuries of other abdominal or thoracic organs, including major vessels [9,10].

Isolated duodenal injuries are particularly rare in blunt abdominal trauma as the duodenum is at a deep and relatively well-protected anatomical site and thus the jejunum is the most injured segment [11]. A 6-year study on blunt duodenal rupture, found the incidence to be 0.2%, with the most commonly injured duodenal segment being D2 at 27%. D3 ruptures accounted for 17% and many of these were associated with other injuries while D4 accounted for 13%. Isolated duodenal injury was noted to be at 1.45% [12,3].

An accepted mechanism for bowel rupture is compression of the fluid-gas filled viscus against the spine, causing tearing of the mesentery. It is also possible that rapid deceleration and inert stress of the small bowel to tethering structures, such as the ligament of Treitz and hepatoduodenal ligament cause rupture at these sites [13].

Duodenal injuries secondary to blunt trauma can range in severity from intramural haematoma to a complete transection and devascularisation of the duodenum, and are graded 1–5 by the American Association for the Surgery of Trauma [14]. Our patient had grade II duodenal injury with a laceration of < 50% of the circumference.

Diagnosis of blunt duodenal injury is often delayed because of its retroperitoneal nature so patients may not present with peritonitis initially unless a very high index of suspicion is kept. It is therefore important to consider both mechanism of injury and also other clinical signs such as tachycardia and raised white cell count as delays in diagnosis and subsequent management have been shown to adversely affect morbidity and mortality [5,3]. Initial clinical changes in isolated duodenal injury may be extremely subtle before the severe peritonitis develops [1]. Although serial determination of serum amylase is better than a single, isolated assay on admission, sensitivity is still low and serial determination involves unnecessary delay in the treatment

Ultrasound and CT scan form the mainstay of initial investigation. Ultrasound is used mainly to demonstrate free intraperitoneal fluid and to screen for any obvious solid organ injury but is an inadequate test for the pancreatico-duodenal area [10]. Ultrasound is reported to have correctly identified patients for laparotomy after blunt abdominal trauma in 100% of 1671 cases [15]. Sensitivity, specificity and accuracy of detecting intra-abdominal injury were 88%, 100% and 99%, respectively. However, the sensitivity for detecting intestinal injury has been reported as 34.7% by sonography alone [16]. CT more clearly demonstrates anatomy and has been reported to be diagnostic of bowel injury in 88% of blunt abdominal trauma cases and so it is the diagnostic test of choice in stable patients with blunt abdominal trauma. The presence of retroperitoneal extra luminal air on CT is an important sign of duodenal injury requiring surgical repair. It is also a useful adjunct in aiding in the differentiation between full thickness rupture that requires surgical intervention and a haematoma which can be managed conservatively. Occasionally it may be negative as it was in our patient or give subtle findings like small amount of unexplained fluid in the abdomen. This can happen if the CT scan is done too early after the injury [3].

The literature suggests that kocherisation (mobilization) of the duodenum should be performed to allow full examination of the duodenum to rule out multiple perforations or rule out significant injury even in the absence of obvious trauma [9,3]. There are multiple ways to repair a duodenal rupture and these are dependent on the severity of the injury right from simple primary closure-duodenorraphy to complex procedures like resection and anastomosis, duodenal diverticulation, pyloric exclusion and pancreaticoduodenectomy.

Isolated duodenal injury is very rare in blunt abdominal trauma and general surgeons should have a high clinical index of suspicion in the initial clinical evaluation of trauma patients and also during trauma related laparotomies.

## Declaration of Competing Interest

All authors: Marilyn Omondi, Irene Mutua and Dan Kiptoon have no conflict of interest.

## Sources of funding

No funding or grant support.

## Ethical approval

Approval for publication of case report titled “Isolated duodenal perforation at D4 following blunt abdominal trauma” was obtained from Kenyatta National Hospital- University of Nairobi Ethics and Research Committee (KNH-UON ERC). The Ref Number is: KNH-ERC/01/PUB/2.

## Consent

Written informed consent was obtained from the patient for publication of this case report. This report does not contain any personal information that could lead to the identification of the patient. A copy of the written consent is available for review by the Editor- in Chief of this journal on request.

## Author contribution

1. Dr Marilyn Omondi - Consultant General Surgeon, Department of Surgery, College of Health Sciences, P.O Box 19676-00202, University of Nairobi, Kenya.

Was involved in the case report conception, management of the patient, drafting the manuscript and its critical revision.

I am the corresponding author.

2. Dr. Irene Mutua - Pediatric Surgery Resident, Department of Surgery, College of Health Sciences, P.O Box 19676-00202, University of Nairobi Kenya. Mobile Phone: 0711261324 Email: immutua@yahoo.com.

Was involved in the case report conception, management of the patient, drafting the manuscript and its critical revision.

3. Dr Dan Kiptoon - Lecturer and Consultant General Surgeon, Department of Surgery, College of Health Sciences, P.O Box 19676-00202, University of Nairobi, Kenya.

Was involved in the case report conception, management of the patient, drafting the manuscript and its critical revision.

## Registration of research studies

This is a case report of a patient we managed and not a human study research.

## Guarantor

Dr Dan Kiptoon.

## Authorship

All authors attest that they meet the current uscript and its critical revision.

3. Dr Dan Kiptoon - Lecturer and Consultant General Surgeon, Department of Surgery, College of Health Sciences, P.O Box 19676-00202, University of Nairobi, Kenya.

Was involved in the case report conception, management of the patient, drafting the manuscript and its critical revision.

## Registration of research studies

This is a case report of a patient we managed and not a human study research.

## Guarantor

Dr Dan Kiptoon.

## Authorship

All authors attest that they meet the current ICMJE criteria for Authorship.

## Provenance and peer review

Not commissioned, externally peer-reviewed.
